# Dermoscopy and Reflectance Confocal Microscopy in the Diagnosis and Management of Nail Fold Squamous Cell Carcinoma

**DOI:** 10.25122/jml-2019-0129

**Published:** 2020

**Authors:** Sara Mazzilli, Terenzio Cosio, Laura Diluvio, Laura Vollono, Salvador Gonzalez, Monia Di Prete, Augusto Orlandi, Luca Bianchi, Elena Campione

**Affiliations:** 1. Dermatologic Unit, University of Rome Tor Vergata, Rome, Italy.; 2. Anatomic Pathology Unit, University of Rome Tor Vergata, Rome, Italy.; 3. Department of Medicine and Medical Divisions, University of Alcalá, Madrid, Spain.

**Keywords:** reflectance confocal microscopy, non-melanoma skin cancer, nailfold squamous cell carcinoma

## Abstract

The management and prognosis of squamous cell carcinoma largely depend on its invasiveness and grade of differentiation. Pigmented nail fold squamous cell carcinoma represents a therapeutic challenge, needing careful treatment to preserve nail function. Here, we report the use of dermoscopy and Reflectance Confocal Microscopy to monitor nail fold squamous cell carcinoma in situ and its response to treatment with topical imiquimod.

## Introduction

Squamous cell carcinoma (SCC) of the nail (NSCC) is a low-incidence malignant tumor affecting this apparatus and is usually slow-growing [1-2]. Its etiology remains unknown, but exposure to ionizing radiations, ultraviolet radiations, and HPV infection are risk factors. To date, there is no complete consensus on the classification, grading, and treatment of NSCC. A classification based on distinct epithelial origin related to different clinical presentations and clinical behavior has been recently proposed, paving the way for standardization in the management of this tumor [3].

NSCC is more frequent on the thumbs of older men and exhibits aggressive clinical behavior, although metastasis is rare. Early signs and symptoms of the disease include onycholysis and erythema, while ulceration is a sign of advanced disease. In particular, pain, swelling, and inflammation are linked to tumor-spreading to the underlying bone [4].

Mounting evidence shows that dermoscopy and reflectance confocal microscopy (RCM) represent useful tools for the diagnosis of SCC and other disorders of the keratinocytes, such as actinic keratosis (AK). RCM exhibits buttonhole vessels, white structureless areas, and dotted or glomerular vessels significantly associated with in situ lesions [5]. On the other hand, dermoscopic criteria of SCC include keratin accumulations, scaling, blood spots, white circles, white structureless zones and perivascular white halos [5]. However, the adherent surface scale sometimes obscures the underlying morphologic features. This is especially true for SCC localized in the periungual area. RCM examination may represent the bridge on the gap between dermoscopic and histologic analyses, becoming a useful tool to evaluate the therapeutic effect of different agents in a non-invasive manner [6].

Here, we report a combined approach, consisting of dermoscopy and RCM, to diagnose a case of NSCC and monitor its response to treatment with topical imiquimod 5% cream.

## Case report

A 46-year-old man presented for evaluation of a nodular lesion on his fifth right fingernail that firstly appeared in February 2018. He did not smoke and had a history of ulcerative colitis, treated with Infliximab since March 2019. Vaccination with nonavalent Gardasil® for HPV was administered one year before this clinical manifestation. Dermatologic examination revealed an exophytic, erythematous mass on the perionychium with focal superficial erosions. Biopsy of the nodular mass was performed, and the histopathological examination showed features consistent with a diagnosis of squamous cell carcinoma in situ (Figure 1 A). In this case, surgical treatment is the first treatment option; however, in order to reduce the field of cancerization and tumor size, and to minimize damages to the surrounding structures, according to the willingness of the patient to preserve the cosmetic and functional integrity of the finger, we prescribed a course of treatment with imiquimod 5% cream daily for eight weeks. (Figure 2 A). In order to evaluate the therapeutic effects of the topical treatment, the patient was monitored by both dermoscopic and RCM examinations before and during therapy, and four weeks and 12 weeks after the treatment. The latest examination of the affected area showed a complete absence of residual tumor.

**Figure 1 A:**
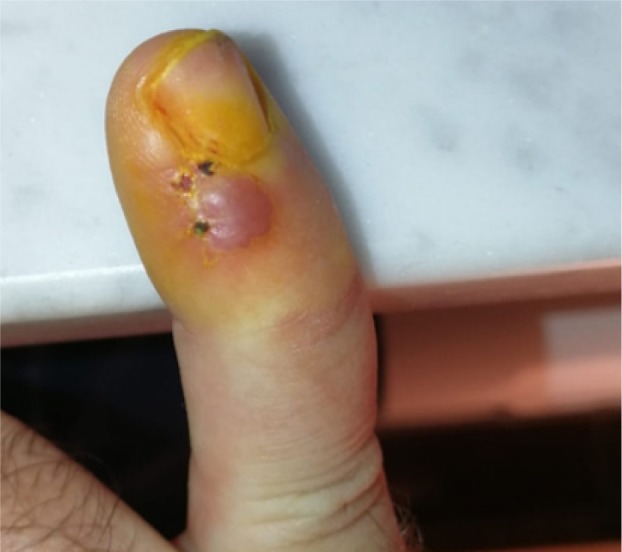
Clinical picture showing the biopsy site and the extent of the lesion.

**Figure 1 B:**
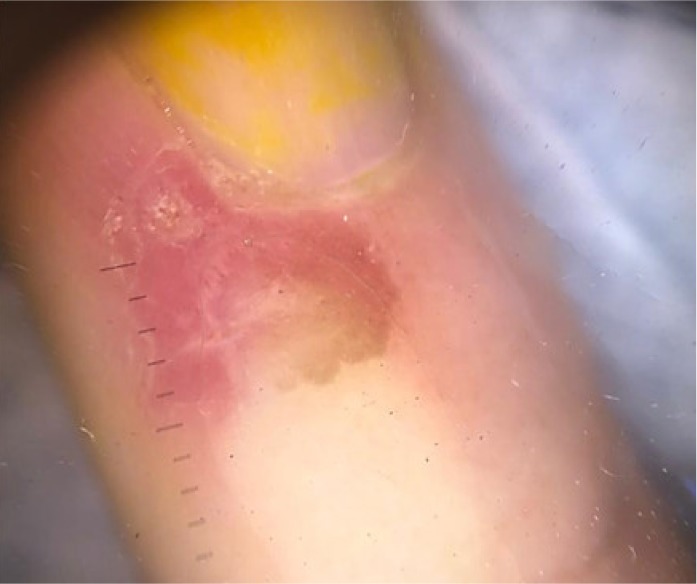
Dermoscopy of pigmented nail fold squamous cell carcinoma shows areas of homogeneous brown pigmentation, pigmented dots or globules, polymorphic vessels.

**Figure 1 C:**
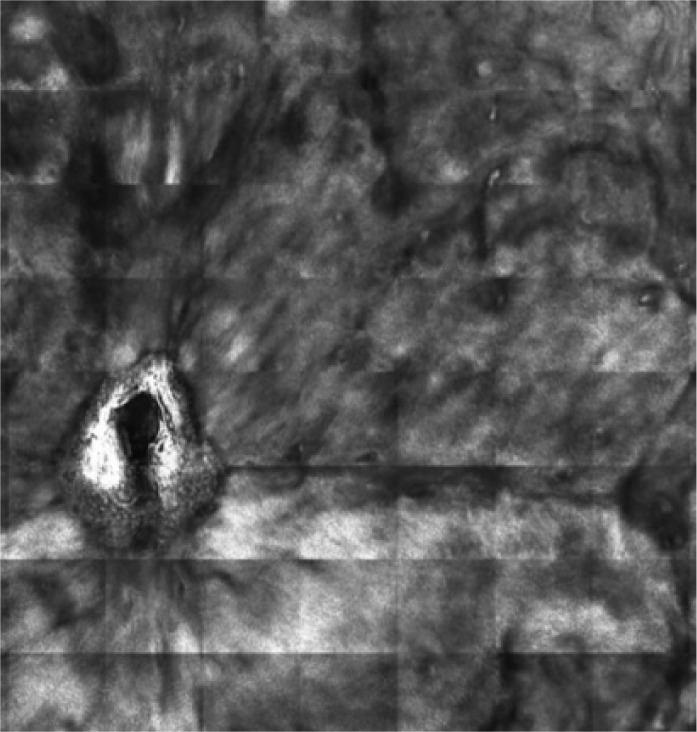
Reflectance confocal microscopy mosaic (3 x 3 mm) at superficial epidermis reveals atypical honeycombing pattern with prominent keratinocytes disarray.

**Figure 1 D:**
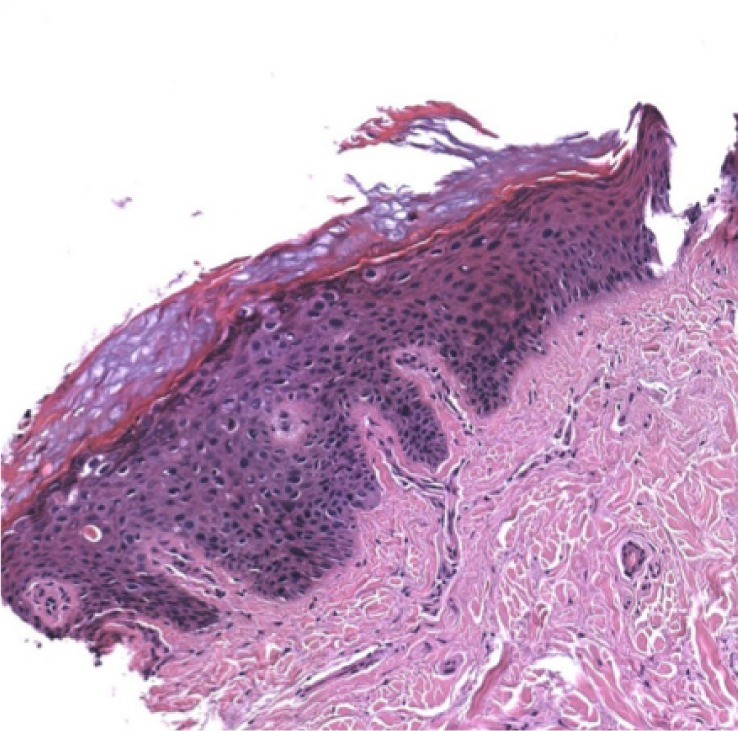
Histopathological examination confirms the diagnosis of squamous cell carcinoma in situ.

**Figure 2 A:**
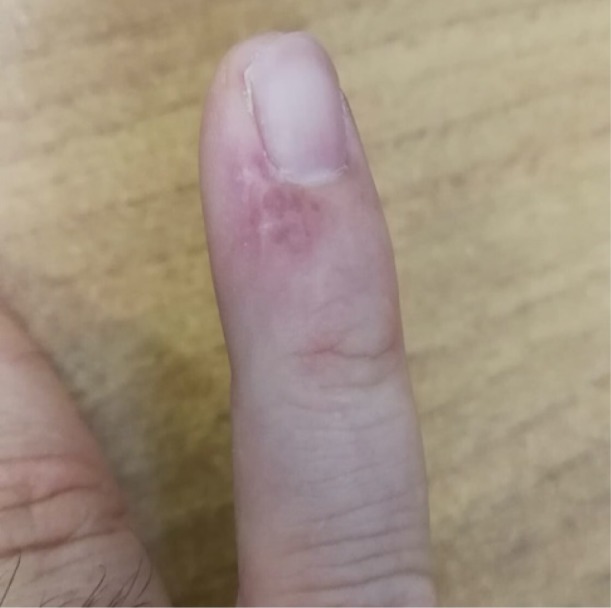
Clinical image four weeks after ending imiquimod therapy shows a regression of the nodular aspect and a reduction of the area involved by the lesion.

## Discussion

After basal cell carcinoma (BCC), SCC should be considered the second most common skin malignancy, and its prevalence is increasing in many countries. It affects every part of the skin and mucosa with squamous epithelium, especially areas exposed to sunlight, such as the face, hands, and arms [7]. Other risk factors for this disease include light skin, aging, chronic ulcers, male gender, AK, and HPV infection [8]. The common high-risk mucosal HPV types (18, 16, and 56) have been frequently found in non-melanoma skin cancers (NMSC) [9]. Although the main cause of NMSC is exposure to UV radiation, cutaneous HPV can also synergistically act with UV radiations in the process of carcinogenesis. In our case, the patient received the HPV vaccine a year before; for this reason, we cannot establish a specific role of HPV in the skin tumor onset. Undoubtedly the patient underwent weekly Infliximab infusion for its inflammatory bowel condition, having a state of periodic immunosuppression. Handisurya et al. reported the correlation between HPV type 26 infection and multiple invasive SCC of the nail in an AIDS patient under highly active antiretroviral therapy [10]. Imiquimod is an immuno-response enhancer able to activate the Toll-like receptor 7 (TLR- 7). Topical imiquimod stimulates the innate and adaptive immune responses, inducing cytokines production [11]. This allows its use for the treatment of a wide variety of benign and malignant skin conditions due to its potential antiviral, antitumoral, and immunoregulatory effects. This topical drug is licensed for adults treatment of external genital warts, superficial BCC, and AK, which can be considered a precursor of SCC [12, 13].

RCM examination has been used to monitor the response of SCC to other non-invasive therapies such as photodynamic therapy [14]. A comprehensive review of the literature did not yield manuscripts in which RCM was used to diagnose or monitor NSCC response to local therapies.

In our case, the dermoscopic examination before treatment revealed the presence of a nodular keratin mass with a white and amorphous central area, white keratin pearls, and hemorrhage associated with peripheral brown amorphous areas (Figure 2B). RCM imaging at baseline showed an atypical honeycombing pattern with keratinocyte disarray, presence of the button-hole sign (round vessel perpendicular to epidermal layer), and atypical round cells (Figure 1B-C). These features are specific criteria for SCC diagnosis [15]. Histopathological examination confirmed NSCC in situ (Figure 1D). RCM imaging four weeks after the end of the imiquimod treatment showed a significant inflammatory reaction that did not allow the visualization of residual tumor masses. Additionally, a high density of stellate shapes was observed (Figure 2 C-D), which may correspond to sweat duct-related structures frequently seen in palms and soles.

**Figure 2 B:**
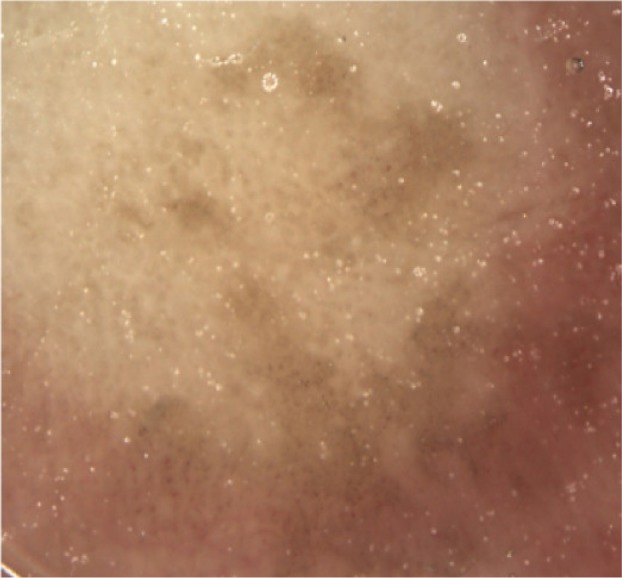
Dermoscopy reveals an irregular pigmentation.

**Figure 2 C:**
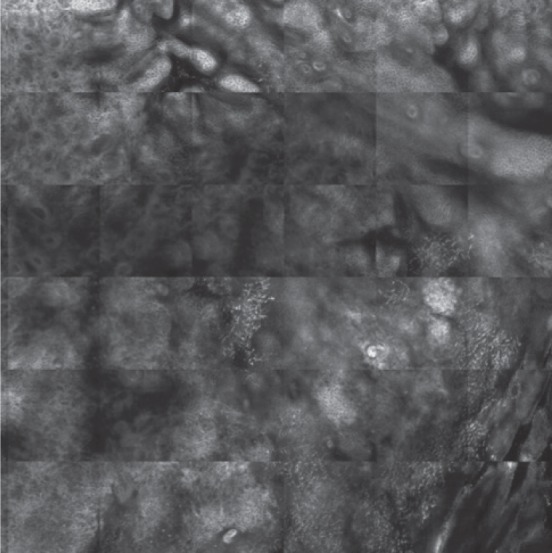
Reflectance confocal microscopy mosaic (3 x 3 mm) shows the presence of area with stellated structures (blue square) in the context of a quite regular honeycomb pattern.

**Figure 2 D:**
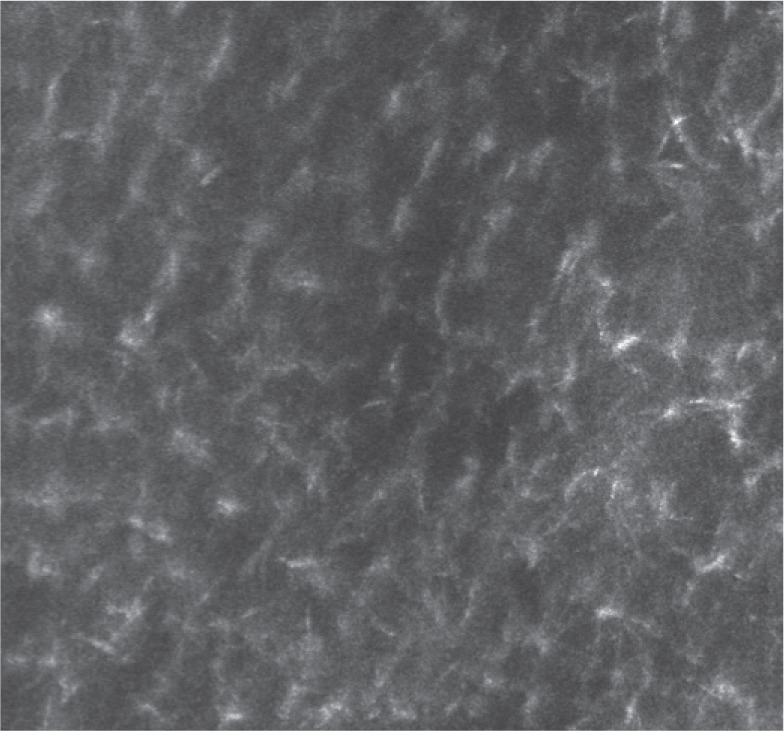
Reflectance confocal microscopy image (0.3 x 0.3 mm) illustrates organized stellated figures that may correspond to sweat-related structures.

Eight weeks after the treatment, dermoscopy revealed the absence of white amorphous areas, white keratin pearls, and hemorrhage with the persistence of brown amorphous areas (Figure 3A). RCM imaging showed a clearly reduced population of stellate figures, in the contest of a more normal-looking honeycombing pattern devoid of evident atypia. (Figure 3 B) Furthermore, RCM examination did not reveal the presence of the button-hole sign and atypical round cells, which are typical features of SCC (Figure 3 C-D)

**Figure 3 A:**
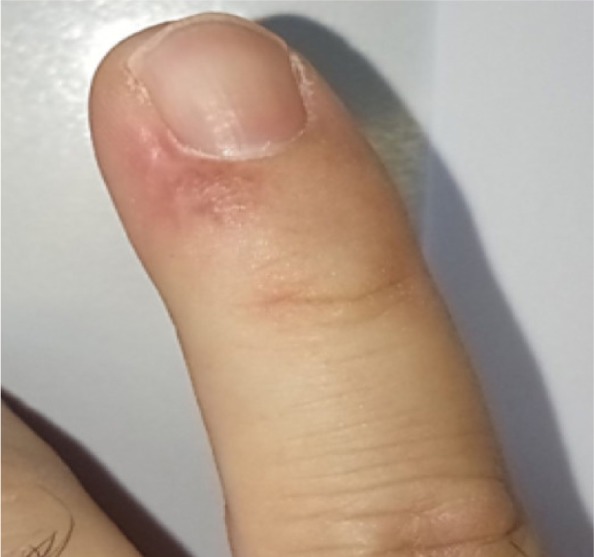
Clinical image eight weeks after the end of the treatment.

**Figure 3 B:**
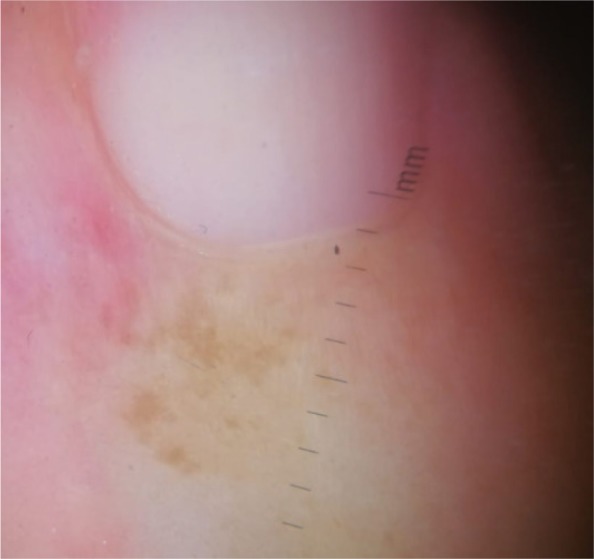
Dermoscopy shows the presence of a mild periungual light-brown pigmentation.

**Figure 3 C:**
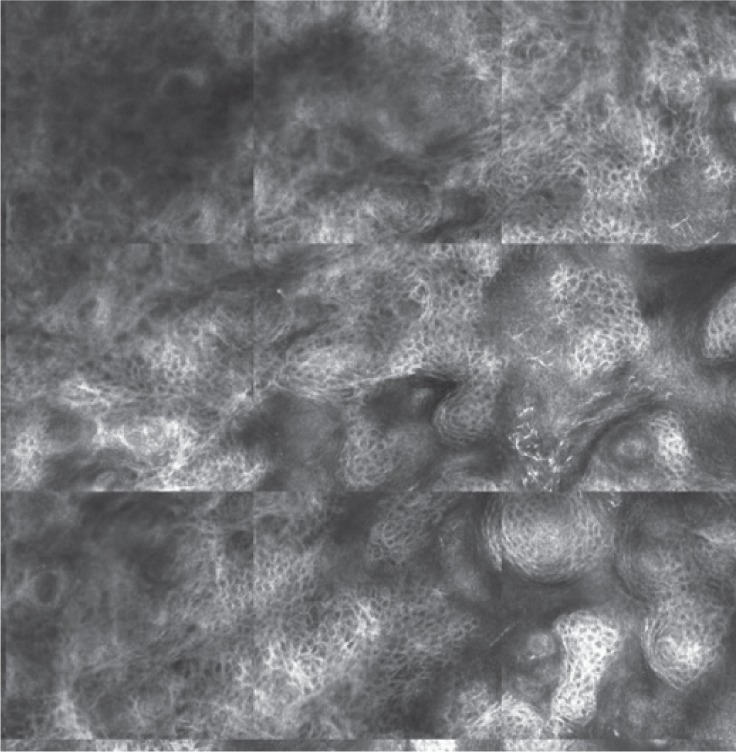
Reflectance confocal microscopy mosaic (1.5 x 1.5 mm) at a superficial level reveals normalization of the epidermis with stellated figures.

**Figure 3 D:**
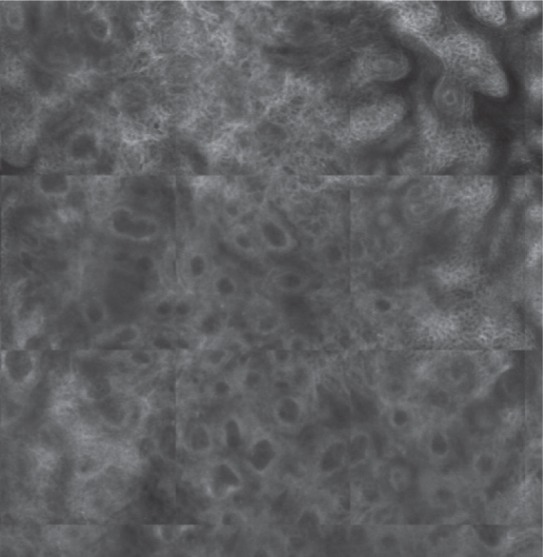
Reflectance confocal microscopy mosaic (1.5 x 1.5 mm) at dermo-epidermal level illustrates bright rounded openings.

## Conclusion

RCM may be a useful tool to diagnose SCC in situ and to monitor the response to non-surgical treatments, by avoiding unnecessary biopsies. This non-invasive examination needs to be performed at least two months after finishing the topical treatment to guarantee the absence of residual tumor due to local activation of the inflammatory pattern. Finally, the data reported so far has been generated based on a single patient and needs further studies.

## Conflict of Interest

The authors confirm that there are no conflicts of interest.
